# 3-(4-Nitro­benz­yl)-4*H*-chromen-4-one

**DOI:** 10.1107/S1600536813006119

**Published:** 2013-03-09

**Authors:** Kaalin Gopaul, Neil Anthony Koorbanally, Mahidansha M. Shaikh, Deresh Ramjugernath, Hong Su

**Affiliations:** a School of Chemistry and Physics, University of KwaZulu-Natal, Durban 4000, South Africa; b School of Chemical Engineering, University of KwaZulu-Natal, Durban, South Africa; c Chemistry Department, University of Cape Town, Rondebosch 7701, South Africa

## Abstract

In the title compound, C_16_H_11_NO_4_, the dihedral angle between the ten-membered chromen-4-one ring system (r.m.s. deviation = 0.0095 Å) and the benzene ring is 86.16 (5)°. In the crystal, mol­ecules are linked into a three-dimensional network by weak C—H⋯O hydrogen bonds. The crystal studied was a non-merohedral twin, with the minor twin component refining to 0.093 (1).

## Related literature

For the preparation, see: Desideri *et al.* (2011 ▶); Valkonen *et al.* (2012 ▶). For related structures, see: Valkonen *et al.* (2012 ▶); Gopaul *et al.* (2013 ▶). For the biological activity of homoisoflavonoids, see: Abegaz *et al.* (2007 ▶).
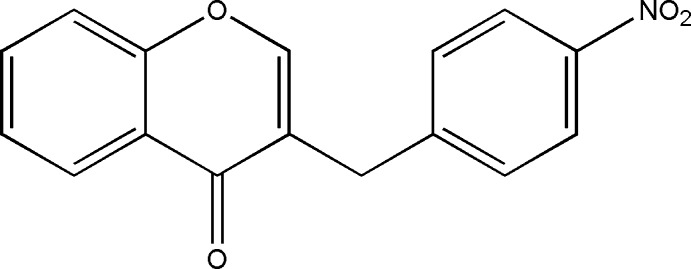


## Experimental

### 

#### Crystal data

C_16_H_11_NO_4_
*M* *_r_* = 281.26
Monoclinic, 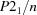

*a* = 4.9246 (9) Å
*b* = 10.0160 (19) Å
*c* = 25.907 (5) Å
β = 92.845 (4)°
*V* = 1276.3 (4) Å^3^
*Z* = 4
Mo *K*α radiation
μ = 0.11 mm^−1^
*T* = 173 K
0.35 × 0.09 × 0.08 mm

#### Data collection

Bruker Kappa DUO APEXII diffractometer
Absorption correction: multi-scan (TWINABS; Sheldrick, 1997 ▶) *T*_min_ = 0.964, *T*_max_ = 0.992
87638 measured reflections
3303 independent reflections
2887 reflections with *I* > 2σ(*I*)
*R*_int_ = 0.063

#### Refinement

*R*[*F* ^2^ > 2σ(*F* ^2^)] = 0.050
*wR*(*F* ^2^) = 0.154
*S* = 1.09
3303 reflections
191 parameters
H-atom parameters constrained
Δρ_max_ = 0.25 e Å^−3^
Δρ_min_ = −0.23 e Å^−3^

### 

Data collection: *APEX2* (Bruker, 2006 ▶); cell refinement: *SAINT* (Bruker, 2006 ▶); data reduction: *SAINT*; program(s) used to solve structure: *SHELXS97* (Sheldrick, 2008 ▶); program(s) used to refine structure: *SHELXL97* (Sheldrick, 2008 ▶); molecular graphics: *ORTEP-3 for Windows* (Farrugia, 2012 ▶); software used to prepare material for publication: *SHELXL97*.

## Supplementary Material

Crystal structure: contains datablock(s) I, global. DOI: 10.1107/S1600536813006119/fj2618sup1.cif

Structure factors: contains datablock(s) I. DOI: 10.1107/S1600536813006119/fj2618Isup2.hkl

Supplementary material file. DOI: 10.1107/S1600536813006119/fj2618Isup3.cml

Additional supplementary materials:  crystallographic information; 3D view; checkCIF report

## Figures and Tables

**Table 1:** Hydrogen-bond geometry (Å, °)

*D*—H⋯*A*	*D*—H	H⋯*A*	*D*⋯*A*	*D*—H⋯*A*
C2—H2⋯O2^i^	0.95	2.38	3.322 (3)	170
C7—H7⋯O3^ii^	0.95	2.56	3.491 (3)	166
C9—H9⋯O4^iii^	0.95	2.42	3.355 (3)	167
C13—H13⋯O3^iv^	0.95	2.53	3.382 (3)	149
C16—H16⋯O2^v^	0.95	2.46	3.378 (3)	163

Symmetry codes: (i) 
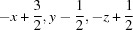
; (ii) 
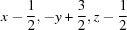
; (iii) 
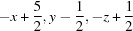
; (iv) 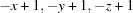; (v) 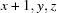.

**Fig. 1.**
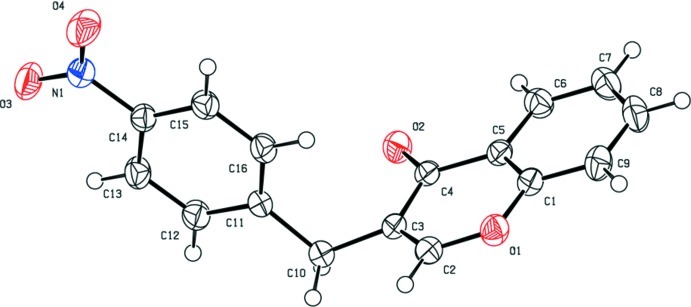
: A view of the molecule with displacement ellipsoids drawn at the 50% probability level and H atoms drawn as circles of arbitary size.

**Fig. 2.**
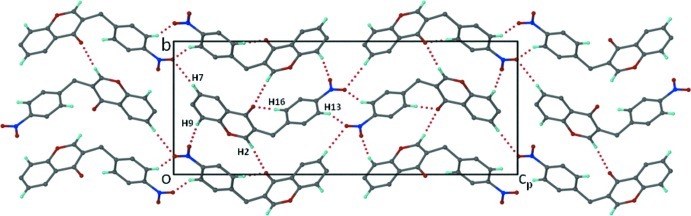
: Projection viewed along the a axis, showing the inter-molecular C—H···O hydrogen bonding network. The H-bond involving H atoms on the molecule of the asymmetric unit were marked for identification. The other H atoms were omitted for clarity. The H-bonds are shown as dotted lines.

## supplementary crystallographic information

### Comment

Homoisoflavonoids are a group of naturally occurring oxygen heterocyclic compounds, related to the flavonoids, which consists of a chromone skeleton with a benzyl or benzylidene group at C-3. In the 3-benzyl-4-chromonanone class of homoisoflavonoid, an extra methylene group exists between the phenyl group and the chromone skeleton. They are commonly synthesized by either the acid or base catalysed condensation of an aromatic aldehyde with chromanone (Desideri *et al.*, 2011, Valkonen *et al.*, 2012). Naturally occurring homoisoflavonoids are normally oxygenated and have shown a wide range of biological activities (Abegaz *et al.*, 2007).

The molecular structure of the title compound is shown in Fig. 1. The dihedral angle between the 10-membered co-planar chromone ring and the nitrated phenyl ring is 86.16 (5)°. In the crystal, the inter-moclecular weak hydrogen bonds C—H···O link the molecules into a three dimentional network, as shown in Fig.2. The details of the hydrogen bonds are shown in Table 1.

### Experimental

A mixture of chroman-4-one (1.02 g, 6.749 mmol), 4-nitrobenzaldehyde (1.22 g, 8.099 mmol) and 10–15 drops of piperidine was heated at 80°C for 12 hrs. The reaction mixture was monitored for completion by thin layer chromatography. Upon completion, the reaction mixture was cooled, diluted with water and neutralized using 10% HCl. To the viscous reaction mixture, 20 ml of ethyl acetate was added. Upon the addition of hexane to the reaction mixture, the homoisoflavonoid precipitated out. The powdered product was filtered, washed with hexane and dried under vacuum. Upon slow evaporation of chloroform, the crystals of the homoisoflavonoid were obtained. (m.p. of 179–180 °C).

^1^H NMR (400 MHz, CDCl_3_) δ: 3.87 (2*H*, s, H-9), 7.38 (1*H*, t, *J*=7.54 Hz, H-6), 7.41 (1*H*, d, *J*=8.60 Hz, H-8), 7.45 (1*H*, d, *J*=8.48 Hz, H-2'/6'), 7.65 (1*H*, td, *J*=8.32, 1.10 Hz, H-7), 7.79 (1*H*, s, H-2), 8.12 (1*H*, d, *J*=8.56 Hz, H-3'/5'), 8.18 (1*H*, dd, *J*=7.98, 0.66 Hz, H-5).

^13^C NMR (100 MHz, CDCl_3_) δ: 31.89 (C-9), 118.15 (C-8), 123.06 (C-3/1'), 123.79 (C-3'/5'), 123.83 (C-4a), 125.32 (C-6), 125.92 (C-5), 129.65 (C-2'/6'), 133.84 (C-7), 146.76 (C-4'), 153.14 (C-2), 156.52 (C-8a), 177.18 (C-4).

### Refinement

The crystal was a non-merohedral twin. Two domains were indexed using *CELL_NOW1* and the intensity data for each domain was then integrated, reduced using the program *SAINT*. The combined data were scaled and absorption correction performed using TWINABS. The structure was solved by direct methods using *SHELXS97* and refined by full-matrix least-squares methods based on F2 using *SHELXL97*. All non-hydrogen atoms were refined anisotropically. All hydrogen atoms were placed in idealized positions and refined with geometrical constraints. The structure was refined to *R* factor = 0.0504, BASF = 0.093 (1) for HKLF5.

### Crystal data

**Table:**

C_16_H_11_NO_4_	*F*(000) = 584
*M**_r_* = 281.26	*D*_x_ = 1.464 Mg m^−^^3^
Monoclinic, *P*2_1_/*n*	Mo *K*α radiation, λ = 0.71073 Å
Hall symbol: -P 2yn	Cell parameters from 3303 reflections
*a* = 4.9246 (9) Å	θ = 1.6–27.4°
*b* = 10.0160 (19) Å	µ = 0.11 mm^−^^1^
*c* = 25.907 (5) Å	*T* = 173 K
β = 92.845 (4)°	Needle, colourless
*V* = 1276.3 (4) Å^3^	0.35 × 0.09 × 0.08 mm
*Z* = 4	

### Data collection

**Table:**

Bruker Kappa DUO APEXII diffractometer	3303 independent reflections
Radiation source: fine-focus sealed tube	2887 reflections with *I* > 2σ(*I*)
Graphite monochromator	*R*_int_ = 0.063
0.5° φ scans and ω scans	θ_max_ = 27.4°, θ_min_ = 1.6°
Absorption correction: multi-scan (TWINABS; Sheldrick, 1997)	*h* = −6→6
*T*_min_ = 0.964, *T*_max_ = 0.992	*k* = 0→12
87638 measured reflections	*l* = 0→33

### Refinement

**Table:**

Refinement on *F*^2^	Primary atom site location: structure-invariant direct methods
Least-squares matrix: full	Secondary atom site location: difference Fourier map
*R*[*F*^2^ > 2σ(*F*^2^)] = 0.050	Hydrogen site location: inferred from neighbouring sites
*wR*(*F*^2^) = 0.154	H-atom parameters constrained
*S* = 1.09	*w* = 1/[σ^2^(*F*_o_^2^) + (0.0779*P*)^2^ + 0.7278*P*] where *P* = (*F*_o_^2^ + 2*F*_c_^2^)/3
3303 reflections	(Δ/σ)_max_ < 0.001
191 parameters	Δρ_max_ = 0.25 e Å^−^^3^
0 restraints	Δρ_min_ = −0.23 e Å^−^^3^

### Special details

**Table:**

Geometry. All e.s.d.'s (except the e.s.d. in the dihedral angle between two l.s. planes) are estimated using the full covariance matrix. The cell e.s.d.'s are taken into account individually in the estimation of e.s.d.'s in distances, angles and torsion angles; correlations between e.s.d.'s in cell parameters are only used when they are defined by crystal symmetry. An approximate (isotropic) treatment of cell e.s.d.'s is used for estimating e.s.d.'s involving l.s. planes.
Refinement. Refinement of *F*^2^ against ALL reflections. The weighted *R*-factor *wR* and goodness of fit *S* are based on *F*^2^, conventional *R*-factors *R* are based on *F*, with *F* set to zero for negative *F*^2^. The threshold expression of *F*^2^ > σ(*F*^2^) is used only for calculating *R*-factors(gt) *etc*. and is not relevant to the choice of reflections for refinement. *R*-factors based on *F*^2^ are statistically about twice as large as those based on *F*, and *R*- factors based on ALL data will be even larger.

### Fractional atomic coordinates and isotropic or equivalent isotropic displacement parameters (Å^2^)

**Table:**

	*x*	*y*	*z*	*U*_iso_*/*U*_eq_	
O1	0.9485 (3)	0.30392 (15)	0.16095 (6)	0.0339 (4)	
O2	0.3498 (3)	0.50967 (15)	0.23136 (6)	0.0355 (4)	
O3	0.8175 (4)	0.63590 (18)	0.49727 (6)	0.0449 (4)	
O4	1.1513 (4)	0.7098 (2)	0.45470 (7)	0.0522 (5)	
N1	0.9499 (4)	0.63878 (18)	0.45839 (7)	0.0328 (4)	
C1	0.8059 (4)	0.4022 (2)	0.13399 (8)	0.0282 (4)	
C2	0.8774 (5)	0.2756 (2)	0.20963 (8)	0.0323 (4)	
H2	0.9747	0.2063	0.2275	0.039*	
C3	0.6800 (4)	0.33749 (19)	0.23490 (7)	0.0281 (4)	
C4	0.5276 (4)	0.44552 (19)	0.20968 (7)	0.0264 (4)	
C5	0.5991 (4)	0.47384 (19)	0.15623 (7)	0.0260 (4)	
C6	0.4653 (5)	0.5734 (2)	0.12658 (8)	0.0333 (5)	
H6	0.3235	0.6233	0.1410	0.040*	
C7	0.5368 (5)	0.6000 (2)	0.07681 (9)	0.0383 (5)	
H7	0.4457	0.6681	0.0572	0.046*	
C8	0.7441 (5)	0.5262 (3)	0.05552 (8)	0.0402 (5)	
H8	0.7927	0.5445	0.0212	0.048*	
C9	0.8801 (5)	0.4268 (2)	0.08354 (8)	0.0364 (5)	
H9	1.0205	0.3767	0.0688	0.044*	
C10	0.6125 (5)	0.2953 (2)	0.28877 (8)	0.0343 (5)	
H10A	0.6971	0.2072	0.2960	0.041*	
H10B	0.4131	0.2836	0.2895	0.041*	
C11	0.7026 (4)	0.39038 (19)	0.33199 (7)	0.0273 (4)	
C12	0.5763 (5)	0.3825 (2)	0.37937 (8)	0.0347 (5)	
H12	0.4330	0.3203	0.3831	0.042*	
C13	0.6553 (5)	0.4630 (2)	0.42049 (8)	0.0341 (5)	
H13	0.5682	0.4569	0.4523	0.041*	
C14	0.8652 (4)	0.55301 (19)	0.41435 (7)	0.0273 (4)	
C15	0.9971 (4)	0.5640 (2)	0.36858 (8)	0.0313 (4)	
H15	1.1411	0.6261	0.3653	0.038*	
C16	0.9136 (4)	0.4816 (2)	0.32739 (8)	0.0309 (4)	
H16	1.0018	0.4879	0.2957	0.037*	

### Atomic displacement parameters (Å^2^)

**Table:**

	*U*^11^	*U*^22^	*U*^33^	*U*^12^	*U*^13^	*U*^23^
O1	0.0369 (8)	0.0348 (7)	0.0303 (7)	0.0077 (7)	0.0045 (6)	−0.0026 (6)
O2	0.0400 (8)	0.0371 (8)	0.0303 (8)	0.0037 (7)	0.0105 (6)	−0.0019 (6)
O3	0.0529 (10)	0.0535 (10)	0.0290 (8)	−0.0090 (8)	0.0089 (7)	−0.0110 (7)
O4	0.0546 (11)	0.0583 (11)	0.0440 (10)	−0.0286 (9)	0.0047 (8)	−0.0118 (8)
N1	0.0365 (9)	0.0334 (9)	0.0284 (9)	−0.0030 (8)	0.0001 (7)	−0.0015 (7)
C1	0.0308 (9)	0.0287 (9)	0.0250 (9)	−0.0015 (8)	0.0003 (8)	−0.0025 (7)
C2	0.0409 (11)	0.0274 (9)	0.0282 (10)	0.0017 (9)	−0.0023 (9)	−0.0006 (8)
C3	0.0368 (10)	0.0251 (9)	0.0222 (9)	−0.0056 (8)	0.0003 (8)	−0.0022 (7)
C4	0.0315 (9)	0.0242 (8)	0.0237 (9)	−0.0051 (8)	0.0034 (8)	−0.0030 (7)
C5	0.0282 (9)	0.0256 (9)	0.0243 (9)	−0.0035 (7)	0.0019 (8)	−0.0025 (7)
C6	0.0362 (10)	0.0321 (10)	0.0316 (10)	0.0035 (9)	0.0018 (9)	0.0024 (8)
C7	0.0425 (12)	0.0404 (11)	0.0318 (11)	0.0005 (10)	−0.0014 (9)	0.0089 (9)
C8	0.0433 (12)	0.0524 (13)	0.0251 (10)	−0.0064 (11)	0.0037 (9)	0.0058 (9)
C9	0.0358 (11)	0.0461 (12)	0.0278 (10)	−0.0012 (10)	0.0067 (9)	−0.0044 (9)
C10	0.0513 (13)	0.0276 (9)	0.0239 (10)	−0.0063 (9)	0.0009 (9)	0.0014 (8)
C11	0.0334 (10)	0.0260 (9)	0.0222 (9)	0.0002 (8)	−0.0005 (8)	0.0026 (7)
C12	0.0384 (11)	0.0375 (11)	0.0286 (10)	−0.0126 (9)	0.0042 (9)	0.0005 (8)
C13	0.0365 (11)	0.0414 (11)	0.0249 (9)	−0.0062 (9)	0.0065 (9)	−0.0004 (8)
C14	0.0290 (9)	0.0290 (9)	0.0238 (9)	0.0007 (8)	−0.0001 (7)	−0.0001 (7)
C15	0.0320 (10)	0.0334 (10)	0.0285 (10)	−0.0060 (8)	0.0017 (8)	0.0022 (8)
C16	0.0337 (10)	0.0340 (10)	0.0253 (9)	−0.0029 (9)	0.0054 (8)	0.0017 (8)

### Geometric parameters (Å, º)

**Table:**

O1—C2	1.356 (3)	C7—H7	0.9500
O1—C1	1.379 (3)	C8—C9	1.385 (3)
O2—C4	1.243 (2)	C8—H8	0.9500
O3—N1	1.227 (2)	C9—H9	0.9500
O4—N1	1.228 (2)	C10—C11	1.519 (3)
N1—C14	1.472 (3)	C10—H10A	0.9900
C1—C5	1.394 (3)	C10—H10B	0.9900
C1—C9	1.397 (3)	C11—C16	1.393 (3)
C2—C3	1.350 (3)	C11—C12	1.406 (3)
C2—H2	0.9500	C12—C13	1.376 (3)
C3—C4	1.453 (3)	C12—H12	0.9500
C3—C10	1.511 (3)	C13—C14	1.387 (3)
C4—C5	1.473 (3)	C13—H13	0.9500
C5—C6	1.403 (3)	C14—C15	1.385 (3)
C6—C7	1.379 (3)	C15—C16	1.394 (3)
C6—H6	0.9500	C15—H15	0.9500
C7—C8	1.396 (3)	C16—H16	0.9500
			
C2—O1—C1	118.18 (16)	C8—C9—C1	118.4 (2)
O3—N1—O4	122.80 (18)	C8—C9—H9	120.8
O3—N1—C14	118.74 (17)	C1—C9—H9	120.8
O4—N1—C14	118.46 (18)	C3—C10—C11	115.84 (17)
O1—C1—C5	121.51 (18)	C3—C10—H10A	108.3
O1—C1—C9	116.75 (18)	C11—C10—H10A	108.3
C5—C1—C9	121.74 (19)	C3—C10—H10B	108.3
C3—C2—O1	125.48 (19)	C11—C10—H10B	108.3
C3—C2—H2	117.3	H10A—C10—H10B	107.4
O1—C2—H2	117.3	C16—C11—C12	118.41 (18)
C2—C3—C4	119.50 (18)	C16—C11—C10	122.68 (18)
C2—C3—C10	121.08 (19)	C12—C11—C10	118.86 (18)
C4—C3—C10	119.42 (18)	C13—C12—C11	121.51 (19)
O2—C4—C3	122.79 (18)	C13—C12—H12	119.2
O2—C4—C5	122.13 (18)	C11—C12—H12	119.2
C3—C4—C5	115.08 (17)	C12—C13—C14	118.41 (18)
C1—C5—C6	118.21 (18)	C12—C13—H13	120.8
C1—C5—C4	120.18 (18)	C14—C13—H13	120.8
C6—C5—C4	121.60 (18)	C15—C14—C13	122.27 (19)
C7—C6—C5	121.0 (2)	C15—C14—N1	119.37 (18)
C7—C6—H6	119.5	C13—C14—N1	118.35 (17)
C5—C6—H6	119.5	C14—C15—C16	118.37 (19)
C6—C7—C8	119.5 (2)	C14—C15—H15	120.8
C6—C7—H7	120.2	C16—C15—H15	120.8
C8—C7—H7	120.2	C11—C16—C15	121.03 (18)
C9—C8—C7	121.2 (2)	C11—C16—H16	119.5
C9—C8—H8	119.4	C15—C16—H16	119.5
C7—C8—H8	119.4		
			
C2—O1—C1—C5	1.8 (3)	C7—C8—C9—C1	−0.3 (4)
C2—O1—C1—C9	−178.58 (19)	O1—C1—C9—C8	−179.1 (2)
C1—O1—C2—C3	−1.4 (3)	C5—C1—C9—C8	0.5 (3)
O1—C2—C3—C4	−0.9 (3)	C2—C3—C10—C11	107.3 (2)
O1—C2—C3—C10	178.39 (19)	C4—C3—C10—C11	−73.4 (3)
C2—C3—C4—O2	−177.5 (2)	C3—C10—C11—C16	−23.1 (3)
C10—C3—C4—O2	3.2 (3)	C3—C10—C11—C12	159.7 (2)
C2—C3—C4—C5	2.6 (3)	C16—C11—C12—C13	0.4 (3)
C10—C3—C4—C5	−176.76 (17)	C10—C11—C12—C13	177.8 (2)
O1—C1—C5—C6	179.24 (18)	C11—C12—C13—C14	−0.1 (3)
C9—C1—C5—C6	−0.3 (3)	C12—C13—C14—C15	−0.3 (3)
O1—C1—C5—C4	−0.1 (3)	C12—C13—C14—N1	−179.9 (2)
C9—C1—C5—C4	−179.63 (19)	O3—N1—C14—C15	174.2 (2)
O2—C4—C5—C1	177.96 (19)	O4—N1—C14—C15	−6.2 (3)
C3—C4—C5—C1	−2.1 (3)	O3—N1—C14—C13	−6.2 (3)
O2—C4—C5—C6	−1.3 (3)	O4—N1—C14—C13	173.4 (2)
C3—C4—C5—C6	178.63 (18)	C13—C14—C15—C16	0.4 (3)
C1—C5—C6—C7	−0.1 (3)	N1—C14—C15—C16	179.96 (19)
C4—C5—C6—C7	179.1 (2)	C12—C11—C16—C15	−0.3 (3)
C5—C6—C7—C8	0.4 (3)	C10—C11—C16—C15	−177.6 (2)
C6—C7—C8—C9	−0.2 (4)	C14—C15—C16—C11	0.0 (3)

### Hydrogen-bond geometry (Å, º)

**Table:**

*D*—H···*A*	*D*—H	H···*A*	*D*···*A*	*D*—H···*A*
C2—H2···O2^i^	0.95	2.38	3.322 (3)	170
C7—H7···O3^ii^	0.95	2.56	3.491 (3)	166
C9—H9···O4^iii^	0.95	2.42	3.355 (3)	167
C13—H13···O3^iv^	0.95	2.53	3.382 (3)	149
C16—H16···O2^v^	0.95	2.46	3.378 (3)	163

Symmetry codes: (i) −*x*+3/2, *y*−1/2, −*z*+1/2; (ii) *x*−1/2, −*y*+3/2, *z*−1/2; (iii) −*x*+5/2, *y*−1/2, −*z*+1/2; (iv) −*x*+1, −*y*+1, −*z*+1; (v) *x*+1, *y*, *z*.
